# Pentoxifylline in Dogs With Osteoarthritis: Comparative Treatment and Efficacy Analysis With Meloxicam

**DOI:** 10.1002/vms3.70427

**Published:** 2025-05-28

**Authors:** Kurtuluş Parlak, Nuriza Zamirbekova Erdogan, Tugba Melike Parlak, Elgin Orçum Uzunlu, Burak Dik

**Affiliations:** ^1^ Department of Surgery, Faculty of Veterinary Medicine Selcuk University Konya Türkiye; ^2^ Department of Pharmacology and Toxicology, Faculty of Veterinary Medicine Selcuk University Konya Türkiye

**Keywords:** dog, meloxicam, pentoxifylline, stifle osteoarthritis

## Abstract

**Background:**

Osteoarthritis is a chronic joint disease that affects dogs and humans alike, with nonsteroidal anti‐inflammatory drugs (NSAIDs) being the primary drug treatment for pain relief. This study hypothesised that pentoxifylline could be a potent therapeutic option for canine osteoarthritis, offering superior benefits compared to meloxicam. The study aimed to mitigate inflammation and degeneration, safeguard joint integrity, and assess pentoxifylline's potential as a safer and more effective alternative to NSAIDs in osteoarthritis management.

**Methods:**

12 dogs with stifle osteoarthritis were divided into two groups: meloxicam (0.1 mg/kg, subcutaneous) and pentoxifylline (10 mg/kg, intramuscular). Clinical, radiological and biochemical examinations were conducted on days 0, 15, 30 and 60.

**Results:**

The pain scores were significantly lower in the meloxicam group on days 30 and 60 (*p* < 0.05). However, both groups had similar scores for other clinical evaluations (*p* > 0.05). Serum C‐reactive protein (CRP) levels were lower in the pentoxifylline group on days 15 and 30, and serum IL‐1β levels were lower on days 15, 30 and 60 (*p* < 0.05). Moreover, cartilage oligomeric matrix protein (COMP) and bone alkaline phosphatase (bALP) levels in the pentoxifylline group showed a significant reduction on day 15 (*p* < 0.05). Pentoxifylline reduced osteocalcin in serum and hyaluronic acid concentrations in synovial fluid on days 15 and 30 (*p* < 0.05). However, the level of cross‐linked C‐telopeptide of type 2 collagen in urine significantly decreased following meloxicam treatment.

**Conclusion:**

Meloxicam relieves pain by protecting joint cartilage, while even low doses of pentoxifylline enhance joint perfusion and combat inflammation. Future research should explore higher pentoxifylline doses and its potential synergy with NSAIDs for superior osteoarthritis management.

## Graphical abstract

12 dogs with bilateral knee osteoarthritis were treated with either meloxicam or pentoxifylline over 60 days. Pentoxifylline showed superior reductions in inflammatory markers (CRP, IL‐1β, IL‐6, TNF‐α) and improved joint biochemical parameters, particularly hyaluronic acid levels and cartilage oligomeric matrix protein (COMP). While both treatments alleviated symptoms, pentoxifylline demonstrated a stronger anti‐inflammatory effect and greater biochemical modulation.

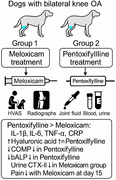

## 1 Introduction

Osteoarthritis is a chronic joint disease that causes pain, loss of function and poor quality of life, often seen in the stifles (Sandersoln et al. 2009; Bland 2015). It typically manifests in older individuals, with environmental, biomechanical, and biochemical factors playing significant roles in its aetiology (Goldring and Goldring 2010). The increasing prevalence of osteoarthritis worldwide, its detrimental effects on quality of life, and the associated high medical costs underscore its critical importance (Murphy et al. 2018). In the United States, approximately one in five adult dogs is affected by osteoarthritis to varying degrees, and it is one of the leading causes of lameness in dogs (Fox 2016).

Changes in joint components such as cartilage, subchondral bone, joint ligaments, and synovium, along with active synovitis and systemic inflammation, are considered responsible for the probable pathogenesis of osteoarthritis (Glyn‐Jones et al. 2015; Mobasheri and Batt 2016). The presence of immune cells such as macrophages and T and B lymphocytes in osteoarthritic joints has been documented (Richard 2011; Mitchell et al. 2013). In osteoarthritic joints, synovial fibroblasts and immune cells release various cytokines, chemokines and matrix metalloproteinases in the state of chondrocytes and synoviocytes (Chow and Chin 2020). This inflammatory and degenerative condition affects the balance of joint anabolism and catabolism (Wojdasiewicz et al. 2014). Some cytokines, such as interleukin‐1 (IL‐1), interleukin‐6 (IL‐6) and tumour necrosis factor‐alpha (TNF‐α), regulate inflammatory pathways in osteoarthritis (Ding et al. 2015).

IL‐1β, which is involved in processes such as cell proliferation, differentiation, and apoptosis, is released from the cartilage tissue, synovium, and synovial fluid in patients with osteoarthritis (Edwards et al. 2011; Kapoor et al. 2011). IL‐1β also stimulates the expression of TNF‐α and its receptor in chondrocytes (Saperstein et al. 2009). The signal transduction initiated by the interaction of TNF‐α and its receptor facilitates inflammatory events through the induction of other cytokines such as IL‐6, matrix metalloproteinases (MMPs), and nitric oxide (NO). This process makes cartilage tissue more susceptible to destructive stimuli and activates osteoclasts, leading to cartilage damage, extracellular matrix degradation, and bone resorption (Fernandes et al. 2002; Kapoor et al. 2011; Chow and Chin 2020). In osteoarthritis, the release of inflammatory mediators also induces the synthesis of C‐reactive protein (CRP) (Wang et al. 2015).

Expression of MMP‐1, which has a collagenase effect, has been associated with the pathogenesis of osteoarthritis, and high levels have been observed in cases of osteoarthritis. MMP‐1, expressed in various joint cells, is a key mediator of ECM degradation in articular cartilage and contributes to irreversible joint destruction in osteoarthritis (Milaras et al. 2021).

The efforts are generally focused on reducing risk factors, physiotherapy, alternative therapy, and pharmacological and surgical treatments to alleviate pain and loss of function and to maintain physical function in later stages (Sanderson et al., 2009; Bland, 2015; Abramoff and Caldera, 2020; Yi et al., 2022). The variability in responses to treatment approaches in osteoarthritis cases makes defining an effective treatment challenging (Sandersoln et al., 2009; Bland, 2015).

Canine osteoarthritis is a complex disease, and veterinarians should optimise the diagnosis and treatment of patients. In this regard, it has been noted that the treatment procedures provided by the COAST Development Group may be applied. However, the guideline indicates that nonsteroidal anti‐inflammatory drugs (NSAIDs) may be used in the treatment of osteoarthritic dogs at stages 2, 3 and 4 (Cachon et al. 2023). Meloxicam, a selective inhibitor of cyclooxygenase‐2 (COX‐2), is used in the treatment of rheumatoid arthritis, short‐term symptomatic treatment of acute exacerbations of osteoarthritis, and symptomatic treatment of ankylosing spondylitis. However, its use is limited in patients with poor cardiovascular health or serious gastrointestinal problems (Khalil and Aldosari 2020).

Pentoxifylline has been reported to be used for the treatment of colitis, bowel anastomosis, venous and gastrointestinal ulcers, various injuries, psoriasis, vasculitis and intermittent claudication, also known as exercise‐induced leg muscle discomfort (Lim et al. 2009; Brasileiro et al. 2013; Ahmadi and Khalili 2016). Pentoxifylline has also been used in dogs for the treatment of atopic dermatitis, contact allergies, tissue necrosis, lick granulomas, vasculitis and other vasculopathies (Marsella et al. 2000; Mueller et al. 2003; Singh et al. 2010; Marsella 2014).

NSAIDs have been associated with adverse effects on the gastrointestinal system, kidneys, and liver during long‐term use. While they typically offer symptomatic treatment by reducing pain and inflammation, NSAIDs are insufficient for slowing the degenerative process of osteoarthritis (such as preserving joint tissue integrity and preventing degenerative changes). Moreover, prolonged NSAID use may lead to reduced efficacy, the development of tolerance, and the emergence of serious side effects in some animals. Additionally, the potential of alternative treatments to contribute to a more comprehensive management of osteoarthritis—by not only modulating inflammation but also enhancing blood circulation, supporting tissue repair, and strengthening antioxidant systems—has driven researchers to pursue alternative therapeutic approaches. (Nagai et al., 2010; Henrotin et al., 2014; Panahi et al., 2016; Zheng et al., 2016).

Pentoxifylline is a synthetic methylxanthine derivative that exerts its effects by inhibiting the phosphodiesterase enzyme (PDE4), subsequently increasing intracellular cAMP levels (Buwalda and Ince 2002; Ahmadi and Khalili 2016). It demonstrates anti‐inflammatory effects by suppressing inflammatory mediators and pro‐inflammatory cytokines (TNFα, TNFβ, IL‐1, IL‐2, and IL‐6) as well as leukocyte functions (Deree et al. 2008; Hussien et al. 2019). Additionally, through its vasodilatory, antithrombotic, and anti‐aggregant properties, it aids in microvascularisation and haemorheology (Fantin et al. 2006; Lim et al. 2009). Pentoxifylline is thought to possess strong therapeutic potential in immunological and inflammation‐based disorders by enhancing blood and oxygen circulation in tissues and its anti‐inflammatory effects. Studies have shown that a few days of pentoxifylline administration contributes to partial ischaemia, anastomosis, collagen synthesis and wound healing (Parra‐Membrives et al. 2007; Carsi et al. 2013; Porwal et al. 2021). In recent studies, pentoxifylline indicates osteogenic and pro‐angiogenic effects and increases osteoblastic activities (Porwal et al. 2021).

While not directly mentioned in the osteoarthritis treatment, properties of pentoxifylline suggest it may have some relevance to osteoarthritis management. Pentoxifylline is known for its immunomodulatory, anti‐inflammatory, antioxidative, angiogenic and vasculogenic effects. In this context, the present study hypothesised that pentoxifylline would be an alternative to meloxicam in dogs with osteoarthritis. This study aimed to reduce inflammation and degeneration, preserve joint structural integrity and establish pentoxifylline as an alternative to standard NSAIDs. The treatments were evaluated based on their effects on joint structure through clinical and radiological examinations. Additionally, their efficacy was assessed through biochemical analyses of joint fluid, serum, and urine, focusing on key markers such as COMP, CRP, IL‐1β, IL‐6, TNF‐α, osteocalcin, BALP, P1NP, type 2 collagen, hyaluronan and MMP‐1.

## 2 Material and Methods

### 2.1 Animal Material

The study was conducted on 12 dogs older than six years who were diagnosed with osteoarthritis of both stifle joints and brought to the Surgery Clinic of the Faculty of Veterinary Medicine at Selcuk University. For inclusion in the study, dogs must not have undergone any surgical procedure within the last 6 months, received any intra‐articular injection in the last 3 months, or been administered any nonsteroidal anti‐inflammatory drugs in the past week. Following clinical examination and radiographic imaging, the animals were determined to be at Grade 2 or 3 according to the COAST guidelines and were included in the study (Cachon et al. 2023). Additionally, the inclusion criteria required that the dogs were not pregnant and did not have any active infections based on the conducted examinations.

### 2.2 Experimental Design and Animal Procedures

12 dogs diagnosed with both stifle osteoarthritis and brought to the Animal Hospital of the Faculty of Veterinary Medicine at Selcuk University were randomly divided into two groups, each consisting of six animals. The experimental design, including study group allocation and sample collection time points, is summarised in Table 1. The dosage selection of the drugs used in the study was determined by modifying previous reference findings.

**TABLE 1:** Study group design and time‐based sample collection.

Day	Group 1	Group 2
**0**.	Blood, joint fluid, radiographic examination, scores, urine sample	Blood, joint fluid, radiographic examination, scores, urine sample
**15**.	Blood, joint Fluid, radiographic examination, scores	Blood, joint fluid, radiographic examination, scores
**30**.	Blood, joint fluid, radiographic examination, scores	Blood, joint fluid, radiographic examination, scores
**60**.	Blood, joint fluid, radiographic examination, scores, urine sample	Blood, joint fluid, radiographic examination, scores, urine sample

**Group 1 (G1) (Standard Treatment Protocol Group, Meloxicam 0.1 mg/kg, subcutaneous route, n = 6)**: The dogs in this group received meloxicam at a dose of 0.1 mg/kg subcutaneously for 60 days (Walton et al. 2014). Blood and joint fluid samples were collected for clinical examination (including gait grading using the Hudson Visual Analog Scale (HVAS‐Hudson Visual Scale) (1–10) and pain assessment tests using the Canine Brief Pain Inventory (1–10)) and radiographic examinations on day 0 (the day before treatment) and on days 15, 30, and 60 post‐treatment. Additionally, urine samples were collected on days 0 and 60.

**Group 2 (G2) (Pentoxifylline Treatment Group, Pentoxifylline 10 mg/kg, intramuscular route, n = 6)**: The dogs in this group received pentoxifylline at a dose of 10 mg/kg intramuscularly for 60 days (Marsella et al. 2000). Blood and joint fluid samples were collected for clinical examination (including gait grading using the HVAS‐Hudson Visual Scale (1–10) and pain assessment tests) and radiographic examinations on day 0 (the day before treatment) and on days 15, 30 and 60 post‐treatment. Additionally, urine samples were collected on days 0 and 60.

### 2.3 Clinical Examination

For the routine clinical examinations of the dogs that constituted the study material, activities such as sitting, walking, climbing and descending stairs, running, and jumping over obstacles were performed. Subsequently, tibial compression and cranial drawer tests were applied to the relevant joints via palpation. Pain assessment tests using the HVAS‐Hudson Visual Scale (1–10) were performed. In the HVAS‐Hudson Visual Scale and the Canine Brief Pain Inventory tests, an increase from 1 to 10 was considered a positive increment. Additionally, the walking and pain assessment tests conducted on days 0, 15, 30 and 60 were performed by the same researcher (veterinarian). Afterward, systemic blood analyses were conducted.

### 2.4 Radiological Examinations

The dogs used in the study underwent radiographic examinations in cranio‐caudal and tibial compression mediolateral positions on the day before the drug applications (day 0) and on days 15, 30 and 60 after the applications, with evaluations recorded. The radiographic images obtained were evaluated by the same veterinarian researcher. The radiographic interpretation was performed according to the Kellgren–Lawrence scoring system (Grade 0: No radiographic findings; Grade 1: Joint space narrowing, possible osteophytic formations; Grade 2: Definite osteophytic formations, severe joint space narrowing; Grade 3: Severe joint space narrowing, sclerosis, severe osteophytic formations, subchondral bone deformity; Grade 4: Severe sclerosis, extensive osteophytic formations, marked subchondral deformity) (Kellgren and Lawrence 1957).

### 2.5 Joint Fluid Analysis

Synovial fluid samples taken from the stifle joints of each dog in the study groups on days 0, 15, 30, and 60 were centrifuged at 2000 rpm for 5 min, immediately frozen and stored at ‐80°C until measurements were performed. The synovial fluid samples collected on the specified days were analysed for hyaluronan (Canine Hyaluronic Acid ELISA Kit, Cat No: E0452Ca, Standard Curve Range: 2–700 ng/ml, Bioassay Technology Laboratory, Shanghai, China) and MMP‐1 (Canine Matrix Metalloproteinase 1 ELISA Kit, Cat No: E0204Ca, Standard Curve Range: 0.05–30 ng/ml, Bioassay Technology Laboratory, Shanghai, China) using dog‐specific commercial ELISA kits according to the procedures specified in the kits. The analyses were conducted using an ELISA reader (Bio‐Tek Instruments Inc., MWGt Lambda Scan 200).

### 2.6 Blood and Urine Analysis

Serum samples separated from blood samples taken from each dog in the study groups on days 0, 15, 30 and 60, and urine samples taken on days 0 and 60, were immediately frozen and stored at ‐80°C until measurements were performed. Urine samples were analysed for Type II collagen (Canine cross linked C‐telopeptide of Type 2 Collagen ELISA Kit, Catalog No: E0441Ca, Standard Curve Range: 3.75–240 ng/ml, Bioassay Technology Laboratory, Shanghai, China) and serum samples for cartilage oligomeric matrix protein (COMP) (Canine cartilage oligomeric matrix protein ELISA Kit, Catalog No: E0392Ca, Standard Curve Range: 1–3000 ng/ml, Bioassay Technology Laboratory, Shanghai, China), C‐reactive protein (CRP) (Canine C‐reactive protein ELISA Kit, Catalog No: E0124Ca, Standard Curve Range: 0.05–30 mg/l, Bioassay Technology Laboratory, Shanghai, China), interleukin‐1beta (IL‐1β) (Canine interleukin 1β ELISA Kit, Catalog No: E0002Ca, Standard Curve Range: 0.2–60 pg/ml, Bioassay Technology Laboratory, Shanghai, China), interleukin‐6 (IL‐6) (Canine interleukin 6 ELISA Kit, Catalog No: E0004Ca, Standard Curve Range: 0.05–1.5 ng/ml, Bioassay Technology Laboratory, Shanghai, China), tumor necrosis factor‐alpha (TNF‐α) (Canine tumor necrosis factor alpha ELISA Kit, Catalog No: E0025Ca, Standard Curve Range: 0.3‐9 ng/l, Bioassay Technology Laboratory, Shanghai, China), osteocalcin (Canine Osteocalcin ELISA Kit, Catalog No: E0146Ca, Standard Curve Range: 0.05–20 ng/ml, Bioassay Technology Laboratory, Shanghai, China), bone alkaline phosphatase (Canine bone alkaline phosphatase ELISA Kit, Catalog No: E0210Ca, Standard Curve Range: 0.05–16 ng/ml, Bioassay Technology Laboratory, Shanghai, China), and the propeptides of Type I collagen (Type I procollagen N‐terminal propeptide, PINP) (Canine procollagen 1 N‐terminal peptide ELISA Kit, Catalog No: E0210Ca, Standard Curve Range: 0.05–16 ng/ml, Bioassay Technology Laboratory, Shanghai, China). These levels were analysed using dog‐specific commercial ELISA kits following the procedures specified in the kits, with measurements performed on an ELISA reader (Bio‐Tek Instruments Inc., MWGt Lambda Scan 200).

### 2.7 Statistical Analysis

The data were analysed using the SPSS 25.0 (SPSS, Inc., Chicago, IL, USA) software. Non‐parametric values (running, radiology, HVAS‐Hudson, etc.) were evaluated as median [interquartile range (IQR)], while parametric values analyzed biochemically (CRP, MMP, osteocalcin, etc.) were evaluated as mean ± standard deviation (SD). For non‐parametric data (data in Table 2), statistical significance between groups was tested using the Mann–Whitney *U* test, and for parametric data (data in Table 3 and figures), it was tested using the independent *T*‐test. Data were considered statistically significant at (*p* < 0.05).

**TABLE 2:** General activation degree, standing, running, climbing, walking, Hvas–Hudson grading and radiological scores in dogs with osteoarthritis at 0 h, 15, 30 and 60 days [Median (interquartile range)].

	0. Hour	15. Day	30. Day	60. Day
**General activation degree**				
Meloxicam	5,0 (1,25)	6,00 (0, 00)	6,50 (1, 50)	7,00 (1,50)
Pentoxifylline	5,00 (2,25)	5,00 (2, 50)	7,00 (2, 50)	7,50 (2,50)
**Standing**				
Meloxicam	5,50 (2, 25)	6,00 (1, 75)	7,00 (1,50)	6,50 (1,25)
Pentoxifylline	5,00 (1, 50)	5,00 (2,50)	7,00 (2,50)	7,50 (4,00)
**Running**				
Meloxicam	5,00 (2,25)	6,00 (1,75)	7,00 (1,50)	6,50 (1,25)
Pentoxifylline	4,50 (2,50)	5,00 (3,50)	7,00 (2,50)	6,50 (4,25)
**Climbing**				
Meloxicam	5,00 (2,25)	5,50 (2,50)	6,50 (1,50)	6,50 (1,25)
Pentoxifylline	4,50 (2,50)	5,00 (3,50)	7,00 (2,50)	6,50 (3,50)
**Walking**				
Meloxicam	5,50 (1,50)	6,00 (1,75)	7,00 (1,50)	6,50 (1,25)
Pentoxifylline	5,00 (1,50)	5,00 (2,50)	7,00 (2,50)	7,50 (3, 25)
**Hvas–Hudson**				
Meloxicam	5,40 (2,43)	6,00 (1,59)	6,50 (1,50)	7,00 (0,80)
Pentoxifylline	5,18 (2,50)	5,22 (3,25)	7,00 (2,50)	7,13 (3,11)
**Radiology**				
Meloxicam	2,00 (0,25)	2,00 (0,00)	2,00 (0,25)	2,00 (0,00)
Pentoxifylline	2,50 (1,25)	2,00 (1,25)	2,00 (1,25)	2,00 (1,25)

No significant differences were found between groups at the specified time points.

**TABLE 3:** Serum cartilage oligomeric matrix proteins (COMP), bone alkaline phosphatase (bALP), osteocalcin, PINP, synovial fluid hyaluronic acid and MMP‐1 levels at 0 h, 15, 30 and 60 days in dogs with osteoarthritis treated with meloxicam and pentoxifylline (Mean ± SD).

	0. Hour	15. Day	30. Day	60. Day
**COMP (ng/mL)**				
Meloxicam	62,44 ± 31,51	45,96 ± 18,58^*^	47,75 ± 24,11	49,04 ± 25,85
Pentoxifylline	53,10 ± 50,42	25,66| ± 7,16	27,81 ± 3,91	29,02 ± 8,39
**bALP (ng/mL)**				
Meloxicam	4,34 ± 4,03	1,95 ± 0,86^*^	1,25 ± 0,68	1,31 ± 0,62^*^
Pentoxifylline	3,80 ± 3,80	0,73 ± 0,23	0,77 ± 0,26	0,68 ± 0,16
**Osteocalcin (ng/mL)**				
Meloxicam	3,98 ± 2,31	5,68 ± 2,65^*^	3,18 ± 1,39^*^	2,96 ± 1,17
Pentoxifylline	5,69 ± 8,11	1,51 ± 0,65	1,75 ± 0,52	2,53 ± 1,00
**PINP (ng/mL)**				
Meloxicam	50,11 ± 27,80	28,30 ± 14,13	20,03 ± 11,47	27,91 ± 14,24
Pentoxifylline	57,26 ± 81,00	17,19 ± 6,19	15,05 ± 1,62	14,78 ± 3,85
**Hyaluronic Acid (ng/mL)**				
Meloxicam	97,07 ± 31,67	103,76 ± 37,76^*^	87,97 ± 35,01^*^	39,82 ± 10,01
Pentoxifylline	76,72 ± 27,10	49,47 ± 16,84	36,56 ± 25,90	40,91 ± 14,32
**MMP‐1 (ng/mL)**				
Meloxicam	5,63 ± 1,76	4,81 ± 0,86	3,19 ± 0,90	3,00 ± 0,67
Pentoxifylline	4,87 ± 2,35	5,30 ± 4,50	2,86 ± 1,60	3,19 ± 1,46

* Meloxicam group values ​​in the same column are statistically different from the pentoxifylline group.

**Abbreviations**: bALP, bone alkaline phosphatase; COMP, cartilage oligomeric matrix protein; MMP‐1, matrix metalloproteinase protein‐1; PINP, procollagen type I amino‐terminal propeptide.

## 3 Results

**FIGURE 1:**
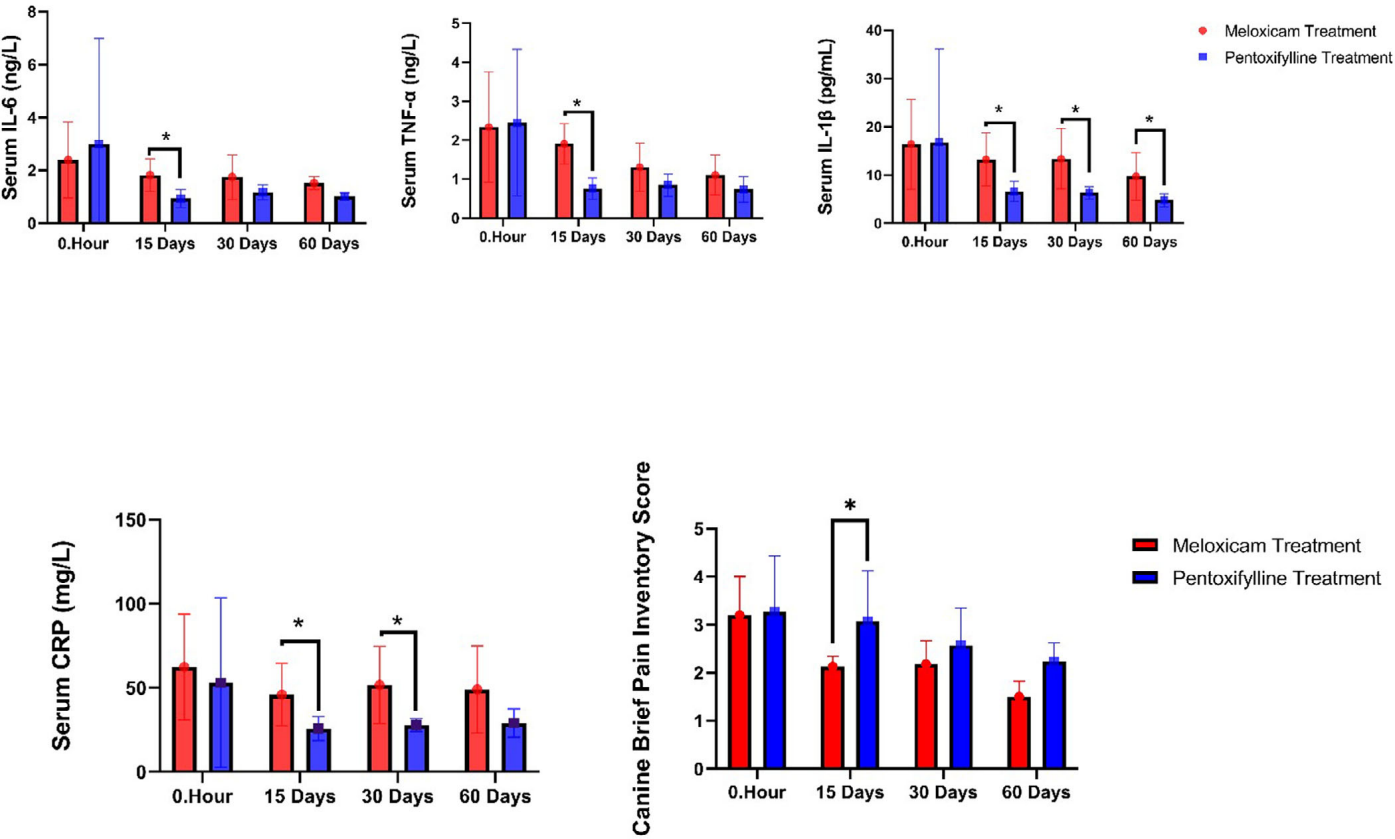
Changes in serum TNF‐α, IL‐1β, IL‐6, CRP, and CBPI levels following pentoxifylline and meloxicam treatment. The graphs display the levels of **(A)** IL‐6, **(B)** TNF‐α, **(C)** IL‐1β, **(D)** CRP and **(E)** Canine brief pain inventory (CBPI) measured in arthritic dogs on days 0, 15, 30 and 60. Data are presented as mean ± standard error of the mean (SD). Groups marked with different letters indicate statistically significant differences (*p* < 0.05).

The effects of meloxicam and pentoxifylline applications on days 0, 15, 30 and 60 on HVAS‐Hudson score and radiological evaluation in dogs with osteoarthritis are presented in Table 2. The scores for pain, general activation, standing up, running, climbing, walking, HVAS‐Hudson and radiological examinations performed before the administration of meloxicam and pentoxifylline were found to be similar between the two groups (*p* > 0.05). On day 15 of the study, it was observed that the pentoxifylline group exhibited lower scores in general activation, standing up, running, climbing, walking, and HVAS‐Hudson assessments compared to the meloxicam group. However, these differences did not reach statistical significance (*p* > 0.05, Table 2). The meloxicam group demonstrated significantly lower pain scores compared to the pentoxifylline group on day 15 (*p* < 0.05, Figure 1).

**FIGURE 2:**
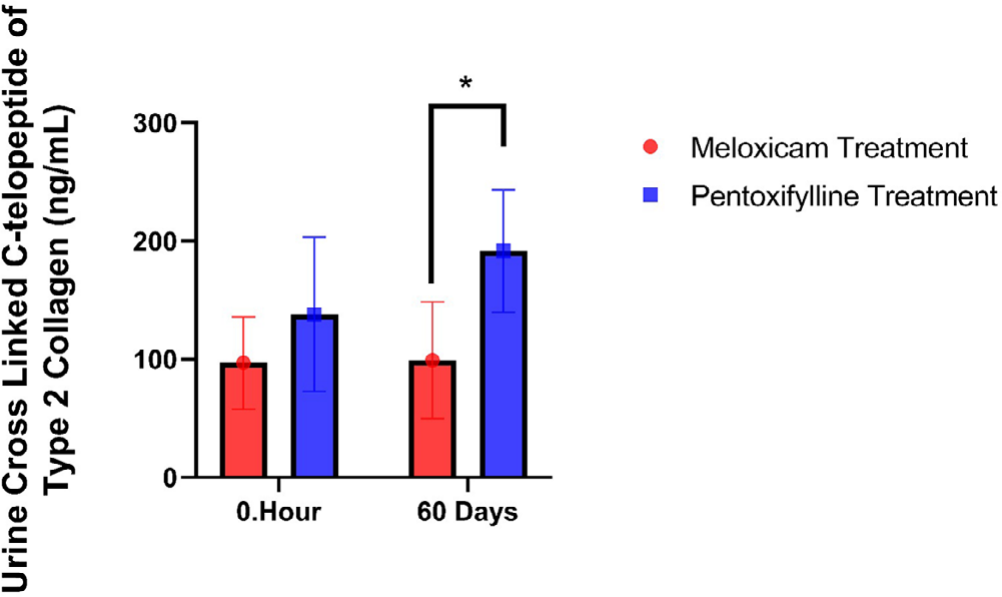
Changes in urinary cross‐linked C‐telopeptide of type II collagen levels following pentoxifylline and meloxicam treatment. The graph presents the levels measured in arthritic dogs on days 0 and 60. Data are expressed as mean ± standard deviation (SD). Groups marked with different letters indicate statistically significant differences (*p* < 0.05).

The effects of meloxicam and pentoxifylline administration on COMP, bALP, osteocalcin, PINP, hyaluronic acid and MMP‐1 levels on days 0, 15, 30 and 60 in osteoarthritic dogs are presented in Table 3. The effects of treatment with meloxicam and pentoxifylline on CRP, IL‐1B, IL‐6 and TNF‐α levels on days 0, 15, 30 and 60 in dogs diagnosed with osteoarthritis are presented in Figure 1. Serum CRP levels in the meloxicam group were found to be higher than in the pentoxifylline group on days 15 and 30 (p<0.05). The lowest CRP level was observed on day 15 in the pentoxifylline group (p<0.05). While both meloxicam and pentoxifylline treatments were observed to reduce serum IL‐1B levels, this reduction was more pronounced in the pentoxifylline group on days 15, 30 and 60 (*p* < 0.05). Compared to the meloxicam group, the reduction in serum IL‐6 levels in the pentoxifylline group on days 15, 30 and 60 was statistically significant only on day 15 (*p* < 0.05). Compared to the meloxicam group, serum TNF‐α levels on days 15, 30 and 60 were lower in the pentoxifylline group, with a statistical difference observed only on day 15 (*p* < 0.05). Although serum COMP levels measured throughout the study were lower in the pentoxifylline group than in the meloxicam group, a statistical difference was observed only on day 15 (*p* < 0.05). Both meloxicam and pentoxifylline applications were found to reduce bone alkaline phosphatase levels measured in serum in dogs with osteoarthritis, but this reduction was statistically significant only in the pentoxifylline group on days 15 and 60 (*p* < 0.05). Serum osteocalcin levels significantly increased with meloxicam treatment on days 15 and 30 (*p* < 0.05), while no statistical difference was observed between the meloxicam and pentoxifylline groups on day 60 (*p* > 0.05). Although serum PINP levels decreased with meloxicam and pentoxifylline treatments in dogs with osteoarthritis, this reduction was not statistically significant (*p* > 0.05). Hyaluronic acid concentrations in synovial fluid were higher in the pentoxifylline group than in the meloxicam group on days 15 and 30 (*p* < 0.05). On day 60, its level was similar in both groups (*p* > 0.05). No statistical difference was observed between the two groups in MMP‐1 values measured from synovial fluid samples on days 0, 15, 30, and 60 (*p* > 0.05). The effects of meloxicam and pentoxifylline on the cross‐linked C‐telopeptide of type 2 collagen on days 0 and 60 are shown in Figure 2. The urinary cross‐linked C‐telopeptide of type 2 collagen levels in the meloxicam treatment group significantly decreased on day 60 (*p* < 0.05, Figure 2).

## 4 Discussion

Osteoarthritis is frequently seen in dogs due to excessive running or exercise, injury, and/or genetic predisposition. Osteoarthritis, also referred to as degenerative joint disease, is a chronic progressive disorder characterised by joint inflammation, pain, stiffness, swelling, and lameness. Degenerative changes in joint structures, including reduced synovial fluid lubrication and altered biochemical composition, lead to cartilage and soft tissue degeneration, bone hypertrophy, and synovial membrane changes. Moreover, mechanical stress disrupts biochemical factors in the affected joints, causing cartilage deterioration (Knazovicky et al. 2016; Anderson et al. 2018; Cui et al. 2020).

Meloxicam is frequently preferred in the treatment of osteoarthritis due to its COX‐2 selective effect and its favourable efficacy in the treatment of locomotor disorders compared to other NSAIDs (Peterson and Keefe 2004; Aragon et al. 2007). The studies conducted on dogs have also shown that meloxicam does not reduce the synthesis of proteoglycans in canine cartilage (Rainsford et al. 1999). In dogs with osteoarthritis, treatment with meloxicam (0.1 mg/kg, oral) for 60 days resulted in a reduction in arthritic lameness and a return to normal daily activities after 30 days. It has been reported that meloxicam is more effective than carprofen in improving conditions and has fewer side effects in dogs with moderate to severe osteoarthritis (Moreau et al. 2003). In another study, meloxicam treatment at a dose of 0.1 mg/kg for 28 days significantly reduced lameness, stiffness, pain, and exercise intolerance, greatly improving the quality of life of the dogs (Doig et al. 2000). Meloxicam (0.1 mg/kg) treated for 4 weeks did not show a significant change in the Canine Brief Pain Inventory (CBPI) score compared to placebo (Panitsillapakit 2021). However, meloxicam treatment in osteoarthritic dogs resulted in a reduction in CBPI score comparable to mavacoxib after 6 weeks (Walton et al. 2014). Meloxicam is effective in reducing pain and restoring clinical joint movement by inhibiting COX‐2, reducing prostaglandin formation, and preventing the degradation of joint cartilage (Peterson and Keefe 2004; Plumb 2011).

Pentoxifylline, used as an alternative treatment in this study, inhibits the enzyme phosphodiesterase, increasing intracellular cAMP levels and consequently exhibiting anti‐inflammatory effects by suppressing inflammation and pro‐inflammatory cytokines (TNFα, TNFβ, IL‐1, IL‐2 and IL‐6) (d'Hellencourt et al. 1996; Marques et al. 1999; Deree et al. 2008; Ahmadi and Khalili 2016). Additionally, its vasodilatory properties, ability to increase microvascularisation and collagen synthesis, and accelerate wound healing are significant features (Fantin et al. 2006; Parra‐Membrives et al. 2007; Lim et al. 2009; Carsi et al. 2013). In a study conducted on mice by Vale et al. (2004), pentoxifylline was observed to reduce the response to pain and joint hyperalgesia in pain and arthritis models, an effect associated with the inhibition of TNF‐α and IL‐1β release. Another study indicated that reduced TNF‐α levels led to a decrease in the expression and secretion of certain matrix metalloproteinases, enzymes regulating extracellular matrix degradation (Babaei and Bayat 2015).

In the current study, the analgesic effect of pentoxifylline was weaker than that of meloxicam according to the CBPI, while no difference was noted in terms of general activation, getting up, running, climbing and walking. Although similar improvements are observed in physical dysfunction due to osteoarthritis, the anti‐inflammatory effects associated with pentoxifylline treatment were more pronounced. Additionally, there was no difference between the two drugs in the HVAS‐Hudson Visual Scale, a walking assessment test, and radiological imaging. It can be speculated that pentoxifylline might exhibit anti‐inflammatory properties by inhibiting the phosphodiesterase enzyme, suppressing pro‐inflammatory cytokines, and increasing microvascularisation and collagen synthesis, thus showing an effect equivalent to meloxicam in the improvement of osteoarthritis. In this regard, it could serve as an alternative to NSAID medications such as meloxicam at higher doses or over a longer duration.

Canine osteoarthritis is a degenerative joint disease marked by pain and bone damage, driven by increased prostaglandins and inflammatory cytokines (Mcreynolds et al. 2019). Proinflammatory cytokines like IL‐1β, TNF‐α and IL‐6 play a key role in cartilage destruction. The imbalance between proinflammatory and anti‐inflammatory cytokines contributes to osteoarthritis development (Liu et al. 2022). CRP is a reliable diagnostic marker of systemic inflammation in dogs for routine use (Christensen et al. 2015). Elevated CRP levels were observed in dogs with idiopathic polyarthritis and decreased significantly after corticosteroid treatment (Ohno et al. 2006). In animal models of arthritis, meloxicam treatment has been found to reduce serum CRP levels and the expression of pro‐inflammatory cytokines such as TNF‐α, IL‐6 and IL‐1β in the joint area. This effect is reported to be achieved by inhibiting the formation of PGE2 from synovial fibroblasts and chondrocytes. It is important in preventing bone‐joint damage and pannus formation (Aslam et al. 2023). In chondrocyte cells obtained from dogs with osteoarthritis, PGE2 release decreased after the third day following meloxicam treatment, but there was no effect on the tissue matrix composition (Budsberg et al. 2013). Meloxicam treatment prevents the reduction of hyaluronan, which plays an important role in the supramolecular organisation of proteoglycan and thus in the biomechanical properties of joint cartilage and reduces joint degeneration (Blot et al. 2000). High‐dose meloxicam treatment, within the first 4 weeks of treatment, prevents the breakdown of type II collagen in serum C‐telopeptide due to its anti‐inflammatory properties and matrix metalloproteinase inhibition, thus contributing to the healing of osteoarthritis (Csifo et al. 2015). In osteoarthritic rats, meloxicam treatment has been observed to reduce levels of IL‐1β, TNF‐α, and COMP, clinically increasing joint healing (Wijekoon et al. 2019). Increased levels of bone alkaline phosphatase (bALP) and osteocalcin are important markers for the healing of osteoarthritis. In dogs with osteoarthritis, the levels of bALP and osteocalcin may increase to ensure bone and cartilage healing (Lavigne et al. 2005). The levels of bALP and osteocalcin have not changed after meloxicam treatment in healthy sheep (Al‐Mashhadane et al. 2014). The detection of urinary CTX II (a biomarker of type II collagen degradation) and procollagen type I N‐terminal propeptide (PINP), markers of cartilage turnover, increases in patients where osteoarthritis is radiologically and functionally identified (Driban et al. 2011). In an experimental osteoarthritis model created in rats, high‐dose meloxicam (1 mg/kg) treatment effectively suppressed inflammation and CTX‐II levels, but low‐dose meloxicam treatment (0.2 mg/kg) was not effective (Csifo et al. 2015). In addition, MMP‐1 levels have returned to normal after the meloxicam treatment in cases of osteoarthritis (Efstathiou and Settas 2017).

Pentoxifylline significantly reduces serum concentrations of inflammatory biomarkers such as TNF‐α, IL‐6 and CRP, and it has vasodilatory properties (González‐Espinoza et al. 2012). Pentoxifylline has been reported to contribute to wound healing by suppressing MMP‐1 expression in the early stages of wound healing (Babaei and Bayat 2015). Pentoxifylline has been observed to accelerate wound healing with its anti‐inflammatory and reperfusion‐enhancing effects by downregulating the expression of TNF‐α, IL‐1β, and IL‐6 (Carsi et al. 2013). It has also been stated that pentoxifylline treatment at various doses improves the healing of segmental cortical bone defects in the radius diaphragm, histologically improves callus formation in bones, and reduces osteoclast levels (Cakmak et al. 2015; Aydın et al. 2011; Vashghani‐Farahani et al. 2019). However, the drug has been interpreted to have a biphasic effect, showing osteogenic effects at low doses (25‐200 mg/kg) and anti‐renewal effects at high doses (300 mg/kg) (Vashghani‐Farahani et al. 2019; Pal et al. 2019).

In the present study, it can be suggested that pentoxifylline may have shown anti‐inflammatory effects by reducing serum levels of inflammatory cytokines such as TNF‐α, IL‐1β, IL‐6 and CRP more than meloxicam, through increased perfusion and inhibition of phosphodiesterase enzyme activity. However, the analgesic efficacy of meloxicam in osteoarthritic dogs, due to COX‐2 inhibition, may have a significant contribution to the reduction of pain. In addition, the decrease in urine CTX‐II levels and the higher levels of bone alkaline phosphatase, hyaluronan, and osteocalcin with meloxicam treatment suggest that cartilage degradation may have decreased, and this treatment may have contributed to the reduction of pain in osteoarthritic animals. However, the absence of these effects in animals treated with pentoxifylline may be dose‐dependent. From this perspective, it can be speculated that pentoxifylline treatment may provide a supportive or alternative effect on meloxicam therapy through a different mechanism of action.

## 5 Conclusion

Early diagnosis and treatment of osteoarthritis are of great importance. In dogs with osteoarthritis, meloxicam therapy may reduce pain by preventing the wear and degeneration of articular cartilage. In contrast, pentoxifylline may inhibit the progression of inflammation by increasing perfusion within the joint. However, it is suggested that pentoxifylline may be more effective in the treatment of osteoarthritis when administered at higher doses.

Future research should investigate the effects of pentoxifylline at higher and different doses and consider combining it with non‐steroidal anti‐inflammatory drugs like meloxicam. This approach could significantly contribute to the development of new alternative treatments for osteoarthritis and the alleviation of its symptoms. In addition, according to the COAST guidelines, new treatment strategies could be developed by combining these two therapies in dogs with advanced‐stage osteoarthritis.

## Author Contributions

Conceptualisation: BD, KP. Formal Analysis: KP, NZE, BD, TMP. Investigation: NZE, TMP, EOU. Methodology: KP, BD. Supervision: KP. Validation: KP, BD. Visualisation: NZE, TMP and EOU. Writing–original draft: KP, BD, NZE, TMP and EOU. Writing–review and editing: KP, BD. Approval of final manuscript: All authors.

## Ethics Statement

The research protocol was reviewed and approved by The Ethics Committee of Selcuk University, Faculty of Veterinary Medicine (Approval date:30/03/2023, Approval number:2023/03) approved the use of the animals for this study and all study protocols.

## Conflicts of Interest

The authors declare no conflicts of interest.

### Peer Review

The peer review history for this article is available at https://www.webofscience.com/api/gateway/wos/peer‐review/10.1002/vms3.70427.

## Data Availability

The data that support the findings of this study are available from the corresponding author upon reasonable request.
